# A snapshot on molecular technologies for diagnosing FAdV infections

**DOI:** 10.3389/fvets.2025.1558257

**Published:** 2025-04-30

**Authors:** Amina Kardoudi, Fellahi Siham, Allaoui Abdelmounaaim, Kichou Faouzi, Ouchhour Ikram, Jackson Thomas, Benani Abdelouaheb

**Affiliations:** ^1^Department of Veterinary Pathology and Public Health, Agronomic and Veterinary Institute Hassan II, Rabat, Morocco; ^2^African Genome Center, Mohammed VI Polytechnic University, Ben Guerir, Morocco; ^3^Milken Institute School of Public Health, George Washington University, Washington, DC, United States; ^4^Medical Biology Department, Molecular Biology Laboratory, Pasteur Institute of Morocco, Casablanca, Morocco

**Keywords:** fowl adenovirus, molecular diagnosis, polymerase chain reaction, real-time PCR, isothermal amplification, CRISPR/Cas13, genotyping, HRM

## Abstract

Fowl adenoviruses (FAdV) are prevalent in chickens worldwide, responsible for several poultry diseases, including inclusion body hepatitis (IBH), hepatitis-hydropericardium syndrome (HHS), and gizzard erosion (GE), which result in significant economic losses in the poultry industry. Consequently, detection and efficient identification of FAdV serotypes are becoming extremely urgent to monitor outbreaks and develop vaccination strategies. Conventional PCR (cPCR) tests, combined with Restriction Fragment Length Polymorphism (RFLP) or sequencing, were developed for FAdV diagnosis. Although these molecular tests have considerably improved the accuracy of FAdV diagnosis compared with conventional methods, certain drawbacks remain unresolved, including lack of sensitivity and post-PCR analysis. Subsequently, advanced molecular technologies such as real-time PCR (qPCR), Loop Isothermal Amplification (LAMP), Cross-Priming Amplification (CPA), Recombinase Polymerase Amplification (RPA), Digital Droplet Polymerase Chain Reaction (ddPCR), Dot Blot Assay Combined with cPCR, Nanoparticle-Assisted PCR (nano-PCR), PCR-Refractory Quantitative Amplification (ARMS-qPCR), CRISPR/Cas13a Technology, and High-Resolution Melting Curve (HRM), have been developed to improve FAdV diagnosis.

## 1 Introduction

Fowl adenoviruses (FAdVs) belong to *Adenoviridae* family, and the aviadenovirus genus, which comprises avian adenoviruses that share a common antigen (1). Based on whole genome analysis and viral neutralization (VN) test, FAdVs are divided into 5 species (A to E) and 12 serotypes (1-8a, 8b-11), respectively (2, 3). Countries such as the United States of America (USA), the European Union (EU), Australia, and Japan have established their classification systems based on local strains. However, this lack of standardization in serotype numbering can lead to confusion and misinterpretation when comparing articles and research results (4). Therefore, the international committee on taxonomy of viruses (ICTV) has published an international classification system that researchers are required to use in their publications (5).

Due to their vertical and horizontal transmission (6–10), FAdVs are widespread throughout the world (11, 12) and are associated with significant economic losses in the poultry industry. Although most infections are subclinical, some FAdV serotypes are associated with impactful poultry diseases such as inclusion body hepatitis (IBH) (13, 14), adenoviral gizzard erosion (AGE) (15, 16), pancreatic necrosis (17), and hepatitis-hydropericardium syndrome (HHS) (18). The latter is particularly worrying due to its association with high mortality rates from 30 to 80% (18–20). Consequently, efficient diagnosis of FAdV has become highly urgent, as well as developing effective vaccination strategies (21).

FAdV diagnosis is initially based on macroscopic features, followed by histological examination (22). Furthermore, electron microscopy enables a direct visualization of icosahedral viral particles in infected tissues, confirming the FAdV diagnosis (3, 23, 24). On the other hand, various serological tests have been widely employed for FAdV detection, including agar gel immunodiffusion (25), double immunodiffusion (26, 27), immunofluorescence (28), counter-immunoelectrophoresis (29), and agar gel precipitation test (30). However, the VN test represents the gold standard for its ability to differentiate between FAdV serotypes (31, 32). Despite this accuracy, the VN test cannot be used for mass detection due to their significant compromises in terms of cost, time, cell culture, and reference strains (32). Various versions of enzyme-linked immunosorbent assay (ELISA) have been developed to overcome these limitations imposed by conventional serological tests (33–36). Although these tests are economical, rapid, and suitable for mass detection, they present many limitations, notably in terms of cross-reactivity and low sensitivity (37).

Compared with other techniques, molecular techniques offer significant advantages in terms of sensitivity, specificity, rapidity, and safety (38). Several reports have employed *in situ* hybridization (ISH) to detect FAdV DNA using specific probes (39–41). Unlike other molecular techniques, these probes can be directly applied to suspected lesions, enabling the confirmation of FAdV involvement. However, this method is no longer widely used today due to the complexity of its application and the availability of more convenient and reliable diagnostic methods. Polymerase chain reaction (PCR) is a widely used technique for the diagnosis of various infections (42). On the other hand, novel versions of PCR have recently been applied for FAdVs diagnosis, including loop-mediated isothermal amplification (LAMP), cross-primed amplification (CPA), recombinase polymerase amplification (RPA), droplet digital polymerase chain reaction (ddPCR), dot blot assay combined with cPCR, nanoparticle-assisted PCR (nano-PCR), PCR-refractory quantitative amplification (ARMS-qPCR), and CRISPR technology. These tests aim to improve the detection, quantification, and genotyping of FAdVs involved in avian diseases. This review covers the molecular methods used for FAdV detection and genotyping, highlighting their role in overcoming the limitations of traditional diagnostic approaches. It also discusses the strengths and drawbacks of these molecular techniques, offering a detailed analysis of their effectiveness and potential challenges.

## 2 Conventional PCR

Initially, cPCR was used for FAdV diagnosis due to its sensitivity and simplicity. Several PCR assays specifically targeting the hexon gene were initially developed for FAdV diagnosis (43–46) (Table 1). The hexon gene is the longest gene in the FAdV genome and encodes a capsid structural protein, specifically the antigenic determinants of either type, group, and subgroup (47). It has 2 functional components: Pedestals regions P1 and P2, conserved between FAdV serotypes (4, 48), and L1-L4 loops that form hypervariable regions (HVR1-4) (49). These HVRs have been identified exclusively in the L1 and L2 regions (43). Except for the L3 region, these loops are surface exposed and interact with the host immune response, making them targeted in taxonomy and FAdV genotyping (4). It has been reported that analysis of the HVR1 region distinguishes between strains of the same serotype from different geographical regions (44).

**Table 1:** Conventional PCR tests used for FAdV detection and associated genotyping techniques.

Technology	Detection section						Genotyping section		
	Forward primer	Reverse primer	Primer sequence 3′ To 5’	Target gene	Product size (bp)	Results interpretation	Genotyping technique	Results interpretation	References
cPCR (Universal Test)	H1		TGGACATGGGGGGCGACCTA	Hexon (FAdV1)	1,219	Detection of 12 serotypes.	RFLP	Differentiation between serotypes except FAV-4 and FAV-5.	(43, 45)
		H2	AAGGG ATTGACGTTGTCCA						
cPCR (Universal Test)	H3		AACGTCAACCCCTTCAACCACC	Hexon (FAdV1)	1,319	Detection of 12 serotypes.	RFLP (HpaII)	Differentiation between FAV-1, FAV-2, FAV-4, FAV-5, FAV-11 and FAV-12, but not for the others.	(43, 45)
		H4	TTGCC TTGGCGAAAGGCG						
cPCR (Universal Test)	Hexon A		CAARTTCAGRCAGACGGT	Hexon (FAdV1)	900	Detection of 12 serotypes.	RFLP (BsiWI, Sty1, Mlu1, Asp1, Bgl1, Sca1)	Successive use of 6 different endonucleases is needed for complete differentiation of 12 FAdV serotypes.	(43)
		Hexon B	TAGTGATGMCGSGACATCAT						
cPCR (Universal Test)	Hexon C		SKCSACYTAYTTCGACAT	Hexon (FAdV-1)	580	Detection of 12 serotypes. Not specific for FAdV	-	-	(43)
		Hexon D	TTRTCWCKRAADCCGATGTA						
cPCR (Universal Test)	MK89		CCCTCCCACCGCTTACCA	Hexon (CELO)	418	Detection of adenovirus from group I, II and III.	-	-	(46)
		MK90	CACGTTGCCCTTATCTTGC						
cPCR (Specific Test)	FibF1		CAGGGTTACGTCTACTCCCC	Short Fiber (FAdV-4)	1,500	Specific detection of FAdV-4.	RFLP (Alu I)	Differentiation between HPS-FAdV-4 and non-HPS-FAdV-4 isolates.	(158)
		FibR1	TTTGTCACGCGGTGGGGAGG						
cPCR (Specific Test)	F1		TCA TGA ACG AGG AGG TTG	Long Fiber (CELO)	2,382	Amplification of FAdV-1 long fiber gene.	RFLP (Hind I)	Differentiation between pathogenic FAdV-1 strains (99ZH) and non-pathogenic FAdV-1 strains (Ote).	(55)
		F2	GTT CAT TGA TGA TAC CCC						
cPCR (Universal Test)	FAVHF		GACATGGGGTCGACCTATTTCGACAT	Hexon (FAdV-10)	731–743	Amplification of FAdVs from HPS-infected birds.	PCR product sequencing + Southern Hybridization.	Confirm the specificity of amplified DNA.	(51)
		FAVHR	AGTGATGACGGGACATCAT						
cPCR (Universal Test)	FAdVFJSN		AATGTCACNACCGARAAGGC	Hexon	830	Amplification of 96 FAdV field strains from chickens in Poland.	PCR product sequencing	Isolats belong to serotypes FAdV-1, FAdV-4, FAdV-5, FAdV-7, FAdV-8a, FAdV-8b et FAdV-2/11 (FAdV-D).	(44)
		FAdVRJSN	CBGCBTRCATGTACTGGTA						
cPCR (Universal Test)	HexF1		GAYRGYHGGRTNBTGGAYATGGG	Hexon (FAdV-1)	800	Amplification of group I, II and III avian adenoviruses. Used to characterize isolates from chickens with HPS in Japan.	PCR product sequencing	Confirmation of serotype 4 from HPS cases.	(159, 160)
		HexR1	TACTTATCNACRGCYTGRTTCCA						
cPCR (Specific Test)	Hex L1-F		ATGGGAGCSACCTAYTTCGACAT	Hexon	590	Amplification of FAdV associated with IBH in broiler chickens in Turkey.	PCR product sequencing	Confirmation of serotype 8b.	(161)
		Hex L1-R	AAATTGTCCCKRAANCCGATGTA						
cPCR (Specific Test)	F-primer		ACAGCCGTGCGCACCAACTGCCCGAAC	Penton (FAdV-4)	498	Specific amplification of FAdV-4 associated with HPS in China.	PCR product sequencing	Confirmation of the FAdV-4	(125, 162)
		R-Primer	CTGCAGATCCTCGTAGGTAATAAC						
Nested PCR (Universal Test)	polFouter		TNMGNGGNGGNMGNTGYTAYCC	DNA Poly	321	Amplification of all serotypes.	PCR product sequencing	Determining, for the first time, the sequence of the gene encoding the DNA polymerase of FAdV-6, −8b, −7, −8a, −2, −3, −6, −1, and FAdV-11.	(55)
		polRinnner	GTDGCRAANSHNCCRTABARNGMRTT						
	polFinner		GTNTWYGAYATHTGYGGHATGTAYGC						
		polRinnner	CCANCCBCDRTTRTGNARNGTRA						
Duplex PCR (Specific Test)	FAdV 1A		TTCGAGATCAAGAGGCCAGT	Hexon (CELO)	178	Specific Amplification of serotypes 1 Sensitivity is 0.0001.	Electrophoresis	The PCR product is 178-bp for serotype 1 and 227 bp for serotype 5.	(53)
		FAdV 1B	GGTCGAAGTTGCGTAGGAAG						
	FAdV 5A		TAACTGCCGTTTCCACATTCA	Hexon (FAdV-5)	227	Specific Amplification of FAdV-5. Sensitivity is 0.0001.			
		FAdV 5B	AGCTGATTGCTGGTGTTGTG						
cPCR (Universal Test)	52K-F		TGT ACG AYT TCG TSC ARA C	52 K + PIII (CELO)	755–794	Detection of 12 serotypes	-	-	(66)
		52K-R	TARATGGCG CCYTGCTC						
cPCR (Specific Test)	F-FAdV-1		ATTTTCAACACCTGGGTGGAGAGCA	Hexon (CELO)	828	Specific amplification of serotype 1	-	-	(52)
		R-FAdV-1	CACGTTGCCCTTATCTTGC						
cPCR (Specific Test)	F-FAdV-4		CCAACGCCACTACCAACT	Hexon (KR-5)	290	Specific amplification of serotype 4	-	-	
		R-FAdV-4	CCAGTTTCTGTGGTGGTTG						
cPCR (Specific Test)	F-FAdV-2		CCCAATATGATTCTACAGTCCA	Hexon (SR-48)	719	Specific amplification of serotype 2	-	-	
		R-FAdV-2	GAGATGGGTATTGTGGGTTCGTATTCGG						
cPCR (Specific Test)	F-FAdV-8a		TAACCCCTATGAGAATACCACT	Hexon (TR-59)	382	Specific amplification of serotype 8a	-	-	
		R-FAdV-8a	ATTGACCGTTCCGTACTCGAT						
cPCR (Specific Test)	F-FAdV-8b		AAGAACGAGGCGCAAAACACAGCTA	Hexon (764)	261	Specific amplification of serotype 8b	-	-	
		R-FAdV-8b	GTCTAACACGTAGTAAGGCGTTGTTCCA						
Nested PCR (Specific Test)	φPCR-F		TGTACGAYTTTGTSCARAC	52 K (FAdV-4)	500	Specific detection of FAdV-4. Detection limit: 10 copies/μL. Same sensitivity than LAMP-LFD.	-	-	(60)
		φaPCR-R	TARATGGCGCCYTGCTC						
	*nPCR-F		GCATAGAGCAGCAGGTAT						
		*nPCR-R	CGAACTCATCCTCCTCTC						
Nested PCR (Specific Test)	φpX-For		CAGGAAGCGTCGCCAACATCAT	X gene (FAdV-9)	440	Specific detection of FAdV-9. More sensitive than cPCR and same detection range than qPCR	-	-	(59)
		φpX-Rev	ACCGTTTCTCCTTCTCCTCGTTGA						
	*pXin-For		CTTACGGGCGGGCGAACAGC		370				
		*pXin-Rev	CGGCACCTGAAACGGGAACC						
Multiplex PCR (Universal Test)	F-FAdV		CAACAGCCTCTCGTACCCAG	Hexon	102	Simultaneous detection of 7 viruses in ducks. LOD: 10^4^ copies/μL. Reproducible. Specific for FAdV.	-	-	(58)
		R-FAdV	CCGATGTAGTTGGGCCTGAG						

*Internal primers; ϕ, External primers; RE, Restriction enzymes; CELO, Chicken embryo lethal orphan; NR, Not reported. N = G/A/T/C, M = A/C, R = A/G, W = A/T, S = C/G, Y = C/T, K = G/T, H = A/C/T, D = A/G/T, B = C/G/T.

Primers for cPCR were designed in both conserved and variable regions on the FAdV hexon gene. The use of universal primers H1/H2, H3/H4, HexonA/HexonB, HexF/HexR, FAdVF JSN/FAdVR JSN, and HexL1-F/HexL1-R, which hybridize to conserved regions on the hexon gene enabling the amplification of L1 hypervariable regions was used for detection of FAdVs. However, To ensure a universal detection, most of these primers are degenerated, including various alternative sequences to cover all minor variations between the 12 serotypes (43). Nevertheless, it was reported that H1/H2 primers failed to amplify FAdV-3 from the supernatant of infected cell cultures (43). Comparison of the sense primer (H1) with the FAdV-3 hexon gene sequence revealed the existence of 3 mismatches located in the last 9 nucleotides on the 3′ end of H1 (nucleotides at positions 11, 14, and 17) (43). The same study showed that the MK89/MK90 primers amplified only FAdV-1 due to the lack of identity of these primers with the other 11 serotypes. This contrasts with the findings of Xie et al. (46), who have reported that MK89/MK90 is a universal primer. Furthermore, the hexon C/hexon D primers are less specific, as they enabled the amplification of the EDS virus, which belongs to group III of avian adenoviruses. This lower specificity is probably associated with the higher degraded level of the Hexon C/Hexon D primers compared to others (43). Consequently, the use of H1/H2, HexonC/HexonD, and MK89/MK90 primers is not suitable for universal detection as they will inevitably lead to false-negative PCR results. These tests are often associated with enzymatic digestion or sequencing for serotype identification. The use of universal hexon A/Hexon B primers followed by sequencing of the product remains the reference technique used for FAdV serotype identification (12). However, a study revealed that Hexon A/Hexon B primers did not amplify FAdV-5 (50), raising questions about their effectiveness for detecting this serotype.

On the other hand, using specific primers targeting hypervariable regions of the hexon gene, in particular, the L1 loop, which shows a higher degree of variability than the L2 loop, FAdV-4 was successfully detected from HHS cases in India by PCR coupled with Southern hybridization (51). Moreover, FAdV-8a, −8b, −1, −2, −4 were detected from IBH, HHS, and AGE cases in Japan using serotype-specific primers targeting specific regions within the hexon gene (52). In addition, primers specific to FAdV-1 and FAdV-5 have been used in duplex PCR for the simultaneous detection of both serotypes in a single reaction. The size of the PCR product differentiates between these 2 serotypes in the case of co-infections. This technique has proved to be a fast, efficient, specific, and highly effective tool for FAdV-5/1 detection (53).

Besides, fiber genes are also used for specific detection of certain FAdV serotypes as they encode type-specific neutralizing epitopes, non-type-specific neutralizing epitopes, and type-specific neutralizing epitopes for the subgenus (54). Primers targeting fiber genes 1 and 2 have been designed to detect FAdV-4 involved in HHS. Moreover, PCR combined with Restriction Fragment Length Polymorphism (RFLP) of the fiber gene, has allowed distinguishing between pathogenic FAdV-1 isolates involved in GE and non-pathogenic isolates from healthy chickens (55). The technique has also been successfully used to differentiate FAdV-4 pathogenic strains from non-pathogenic strains isolated from healthy chickens in Japan, India, and Pakistan.

Compared to Uniplex PCR, Multiplex PCR (m-PCR) offers significant advantages, such as time savings and the ability to diagnose multiple viruses in a single reaction, making it an effective method for rapid diagnosis of mixed infections (56, 57). In this context, an m-PCR test was developed for the simultaneous detection of 7 viruses causing significant economic losses in the poultry industry, including FAdVs (58). Specific primers were designed for each virus, and the tests for specificity, sensitivity, reproducibility, and repeatability were conducted. The m-PCR test showed no cross-reactivity between these 7 viruses or with other common duck pathogens. In addition, the test was able to detect co-infection with several viruses in clinical samples. However, the assay is not highly sensitive, with a detection limit of 10^4^ copies/μL, raising questions about their effectiveness for FAdV detection.

A nested PCR targeting X gene and 52 k gene with 2 successive cycles using PCR-F/PCR-R as external primers in the first amplification cycle and nPCR primers (nPCR-F/nPCR-R) in the second cycle has been developed for specific detection of FAdV-9, and FAdV-4, receptively (59, 60). This method enhances both specificity and sensitivity, with sensitivity being 100 to 1,000 times greater than conventional PCR. Other cPCR tests targeting the penton gene and the gene encoding for DNA polymerase have also been developed (61).

Although conventional PCR targeting specific genes, such as hexon gene and fiber gene, is considered as an efficient, specific, and reliable tool for FAdVs diagnosis, its considered a not very-sensitive tool. Moreover, these tests require post-PCR steps, including RFLP, Electrophoresis, PCR product sequencing, and interpretation of sequencing results, which increases the cost, time, complexity of the analysis, and the risk of contamination. Additionally, conventional PCR cannot quantify the virus, which is necessary for evaluating the effectiveness of infection control and surveillance measures. Therefore, real-time PCR tests have been developed later to overcome these limitations.

## 3 Real-time PCR

Real-time PCR, also known as quantitative polymerase chain reaction (qPCR), represents a significant advance in molecular biology, offering a powerful molecular diagnostic tool for the detection of various human and animal pathogens (62–64). The method is widely adopted due to its high sensitivity, simplicity, reproducibility, and specificity (65). Studies have suggested that it is ten times more sensitive than cPCR, making it extremely valuable in molecular diagnosis (66). qPCR also offers the possibility of performing real-time quantification, making it an invaluable tool in biomedical research, genetic studies, and diagnostic applications where high accuracy and sensitivity are crucial (67, 68).

Real-time PCR method using SYBR Green, a fluorescent dye that binds to double-stranded DNA emitting detectable fluorescence, was initially developed to detect and quantify FAdV-9 genome in various tissues (59). In this assay, a region located at the right end of the FAdV-9 genome, corresponding to ORF 20A, was used as a target (Table 2). However, the qPCR assay was not specific to FAdV-9 since it also detected other serotypes, such as FAdV-1, FAdV-2, FAdV-8, and FAdV-10. This suggests that the selected region is not specific for FAdV-9, and other regions should be examined. In terms of sensitivity, the test showed a sensitivity of 9.4 copies/μL, which is comparable to nested PCR and 100 times more sensitive than conventional PCR (59).

**Table 2:** Real-time PCR tests used for FAdV detection.

Real-time PCR technology	Test objective	Forward primer	Reverse primer	Probe	Sequence 5′ to 3′	Target gene	Product size	Test performance	References
Real-time PCR with (Syber Green)	Detection of 12 FAdV serotypes.	52 K-fw		-	ATG GCK CAG ATG GCY AAG G	52 K	176	LOD: 6.73 copies/μL Efficiency: 98%. R^2^: 0.999. Universal detection of FAdV. Specific detection of FAdVs only.	(66)
			52 K-fw		AGC GCC TGG GTC AAA CCG A				
Real-time PCR with (Syber Green)	Specific detection of FAdV-9 serotype.	FAdV-9 F		-	ATGGTGTTCTATTGGACGCA	ORF20A	114	LOD: 9.4 copies/μL. Efficiency: 100 R^2^: 1 Not specific for FAdV-9.	(59)
			FAdV-9 R		TGTTTGGATGTTGCACCTTT				
Real-time PCR with (TaqMan)	Specific detection of FAdV-4	FAdV-4 F			TTACGCTTACGGTGCCTACGT	Hexon	87	LOD:10 copies/μL R^2:^ 0.999 Efficiency: 94.9% No cross-reaction with FAdV serotypes. Intra-assay ct variation: 0.22–0.32%. Inter-assay ct variation: 0.74–1.15%;	(72)
			FAdV-4 R		CCGCGTTATTCATGATCCAGTA				
				FAdV-4 S	CGACGGTTCCCAGTCCCTCACG				
Real-time PCR with (TaqMan)	Specific detection of FAdV-8b serotype.	FAdV-Hex_143F			GTTAGACACCACCGCACAGA	Hexon	166	LOD: 0.001 ng/μL Efficiency: 96% R^2^: 0.997	(71)
			FAdV-Hex_143R		GTCACGGAACCCGATGTAGT				
				FAdV-Hex_143_Probe	FAM-CCCTCCTTCTGAGTACGGAGAG-BHQ1				
Real-time PCR with (Syber Green)	Specific detection of FAdV-8.	FAdV-8 F		-	AAATGGTAAACGCGTGGGATC	ORF-1 A/B	NR	Specific for serotype 8.	(163)
			FAdV-8R		TTCTCCGTCTCCGATCTGG				
Real-time PCR with (TaqMan)	Specific detection of FAdV-8a.	FAdV - 8aF			GACAGAGGTCCTTCCTTCAA-	Hexon	NR	LOD: 8 copies/μL Efficiency: 95,1% R^2^: 0.997	(70)
			FAdV - 8aR		TCAGGCTATCGGTAAAGTCC-				
				JSNRT/8a/E	AATCCCTACTCGAACACCCC				
Real-time PCR with (TaqMan)	Specific detection of FAdV-1	FAdV - 1 F			TTCGAGATCAAGAGGCCAGT	Hexon	NR	LOD: 8 copies/μL R^2^: 0,991, Efficiency: 95,03%	(70)
			FAdV - 1 R		GGTCGAAGTTCGTAGGAAG				
				JSN RT1/A	AATCCCTACTCCAACACCCC				
Real-time PCR with (TaqMan)	Detection of 12 FAdVs serotypes.	FAdV RT-PCR Fw			CACAACGTCTGCAGATCAGATTC	Hexon	74	Sensitivity: 0.1 fg of DNA.	(101)
			FAdV RT-PCR Rev		GCGCACGCGATAGCTGTT				
				FAdV-Probe	FAM-ACCCGATCCAGACGGATGACACG-TAMRA				
Multiplex PCR	Simultaneous detection of 7 viruses in ducks.	F-FAdV			CAACAGCCTCTCGTACCCAG	Hexon	102	LOD: 10^4^ copies/μL. Reproducible Specific	(58)
			R- FAdV		CCGATGTAGTTGGGCCTGAG				
MRT-qPCR Assay	Simultaneous detection of 6 avian viruses.	F-FAdV			CCCACCACCTCCCATCT	NR	162	Efficiency: 103,54. R^2^: 0,99. Sensitive Repeatable	(69)
			R-FAdV		TGAGCGTAAACCGTCCC				
				Probe	FAM-TCTGCCCTCCCCAGCCTCCATCT-BQ1				

NR, Not reported; FAM, 6-carboxyfluorescein; BHQ-1, Black Hole Quencher; TAMRA, Tetramethylrhodamine; LOD, limit of detection; R^2^, Correlation Coefficient. N = G/A/T/C, M = A/C, R = A/G, W = A/T, S = C/G, Y = C/T, K = G/T, H = A/C/T, D = A/G/T, B = C/G/T.

Subsequently, a universal SYBR Green-based qPCR test was developed by targeting a conserved region of the 52 K gene. This assay demonstrated high sensitivity and specificity, enabling precise detection and quantification of 5 FAdV species (FAdV-A to FAdV-E) with a detection limit of 6.73 copies/μL of FAdV DNA using standards and control vectors. To establish the standard curve, different regions of FAdV genomes are isolated, meticulously prepared, and cloned into plasmid vectors. The concentration of the plasmid DNA is determined using spectrophotometry, and the number of DNA copies is calculated according to the following formula: [(g/μL of DNA)/(length of the plasmid in base pairs × 660) × 6.022 × 10^23^]. The plasmid DNA is then diluted to several concentrations and used to establish the standard curve, which is included in each qPCR reaction (69). qPCR using SYBR Green represents a simple, sensitive, and cost-effective quantitative approach. However, SYBER Green can bind to non-specific products or contaminants, leading to false-positive results.

Consequently, analysis of melting curves and negative controls is required to interpret qPCR results correctly. Other real-time PCR methods, using fluorogenic probes complementary to the target sequences and doubly labeled with a fluorophore and a quencher, have been developed for specific detection of certain FAdV serotypes, such as FAdV-4, FAdV-8b, FAdV-8a, and FAdV-1 (70–72). The use of specific primers that hybridize to type-specific regions allows qPCR to simultaneously perform real-time molecular detection, quantification, and typing in a single reaction. Notably, TaqMan-based qPCR demonstrated superior efficiency compared to SYBR Green-based qPCR, while both methods exhibited similar sensitivity (72).

Recently, a multiplex real-time PCR has been developed, called multiplex reverse transcription real-time quantitative PCR (MRT-qPCR) (69). This technique was designed to detect co-infection of 6 vertically transmitted or immunosuppressive avian viruses, including Marek’s Disease Virus (MDV), Reticuloendotheliosis Virus (REV), Avian Reovirus (ARV), Chicken Infectious Anemia Virus (CIAV), Infection Bursal Disease (IBD), and FAdVs. Six specific probes were designed, each complementary to one virus, and labeled with a unique fluorophore, allowing differentiation of the signal emitted by the 6 probes during the qPCR reaction. A series of validation and optimization tests were carried out, confirming the high specificity, sensitivity, and repeatability of the MRT-qPCR assay. These characteristics ensure the reliability and relevance of this method in diagnosing viral co-infections in poultry, making it an excellent first-line screening tool for a wide range of viruses before moving on to more genus-specific tests.

## 4 Recent breakthroughs in molecular diagnostics of FAdV infections

Although molecular diagnostic tests, such as conventional PCR have considerably improved diagnostic accuracy compared with traditional methods, certain drawbacks linked to operational complexity remain unsolved. Thus, to improve FAdVs detection in terms of sensitivity, cost, and process time, other advanced molecular tests have been developed.

### 4.1 Loop-mediated isothermal amplification

The LAMP technique was first described by Notomi et al. (73). Since then, it has attracted significant interest due to its simplicity and rapidity compared to other amplification methods. Unlike PCR, which requires a series of temperature cycles, LAMP is an isothermal amplification method that proceeds at a constant temperature between 60°C and 65°C, eliminating the need for a thermocycler (73). The LAMP reaction involves 2 external primers (F3 and B3) and 2 pairs of internal primers (FIB and BIP). One or 2 additional primer pairs, known as ‘loop primers’, can be incorporated to accelerate the reaction and improve its sensitivity (74). The LAMP technique consists of 2 significant steps: synthesis of the initiator serving as a template, followed by cyclic amplification, which produces a DNA mixture of stem ring DNA and cauliflower DNA of different sizes. This method can also be applied to RNA by Reverse Transcription-LAMP (RT-LAMP) (75). Amplification products can be detected by several techniques, including agarose gel electrophoresis, which produces multiple ladder bands, or by real-Time turbidity measurement due to the formation of manganese pyrophosphate precipitates. The addition of hydroxy naphthol blue, calcein, or SYBR Green to the reaction system also offers colorimetric detection of the product (74).

**Figure 1:**
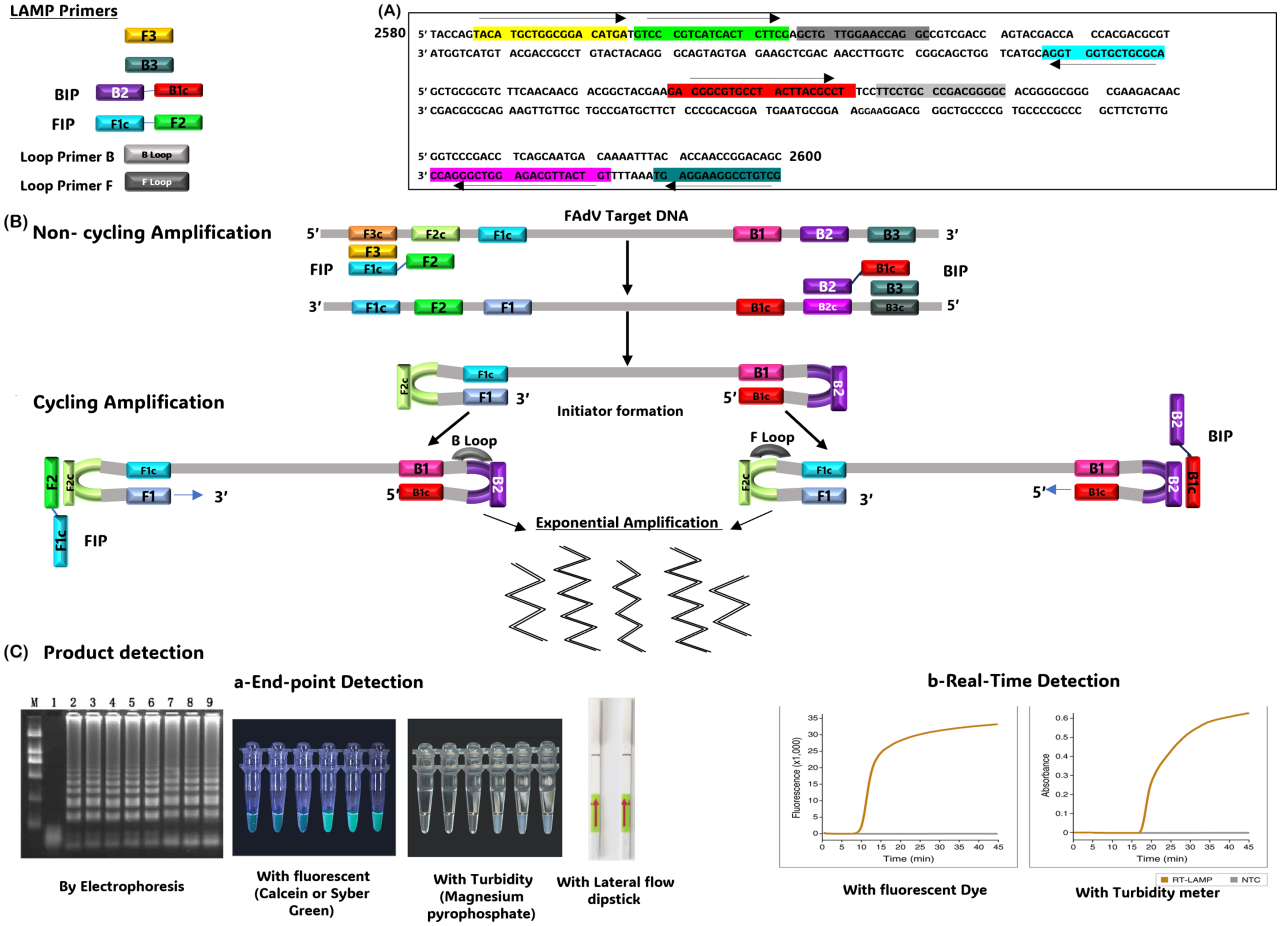
**(A)** Position of the LAMP primers on the FAdV target gene. **(B)** Schematic representation of the Loop-Mediated Isothermal Amplification (LAMP) technique. **(C)** Methods used to detect the LAMP product, including End-point detection (using electrophoresis, addition of fluorescent substrates, turbidity measurement, or lateral flow dipstick or (real-time detection)). The gel image presented in this figure are adapted from previous work (81) and are included to ensure the figure is comprehensive and meaningful.

LAMP assay has been successfully employed to detect many avian viruses, including Infectious Bronchitis Virus (IBV) (75), Chicken Anemia Virus (76), Avian Influenza Virus (AIV) (77), Newcastle Disease Virus (NDV) (78), IBDV (79), and Marek’s Disease Virus (MDV) (80). Recently, a LAMP assay was specifically developed and optimized for FAdVs detection (81). Based on an analysis of their Hexon genes, 4 primer pairs were designed for a conserved region (Figure 1). The LAMP reaction was performed in a water bath at 63°C for 60 min. The addition of SYBR Green fluorescent dye to the reaction gave positive samples a greenish color under ultraviolet (UV) light. Additionally, the formation of white sediment due to precipitated pyrophosphate was a distinctive feature of positive samples. The test shows a detection limit of 238 copies/μL.

To improve the LAMP sensitivity, a real-time LAMP assay has been developed for specific FAdV-4 detection (82). The Mg2P2O7 precipitate produced during the reaction is detected by measuring reaction turbidity every 6 s using a real-time turbidimeter. The assay shows a detection limit of 75 copies/μL of FAdV-4 DNA. Subsequently, a new version of LAMP coupled with lateral flow dipstick (LAMP-LFD) was developed for rapid and specific detection of fowl Adenovirus serotype 4 (60). This test can be completed in 60 min at 65°C, with a detection limit of 10 copies/μL of FAdV-4 DNA, making it more sensitive than real-time LAMP (75 copies/μL) (82), and 1,000 times more sensitive than conventional PCR. However, it has the same detection limit as Nested PCR (60), and specific qPCR (72). Although it remains less sensitive than universal qPCR assay (6.9 copies/μL) (66), it can potentially require less than half time and reagents. These advantages enable LAMP-LFD to be applied in resource-limited areas, such as small farms and basic veterinary laboratories (83).

### 4.2 Cross-priming amplification method

**Figure 2:**
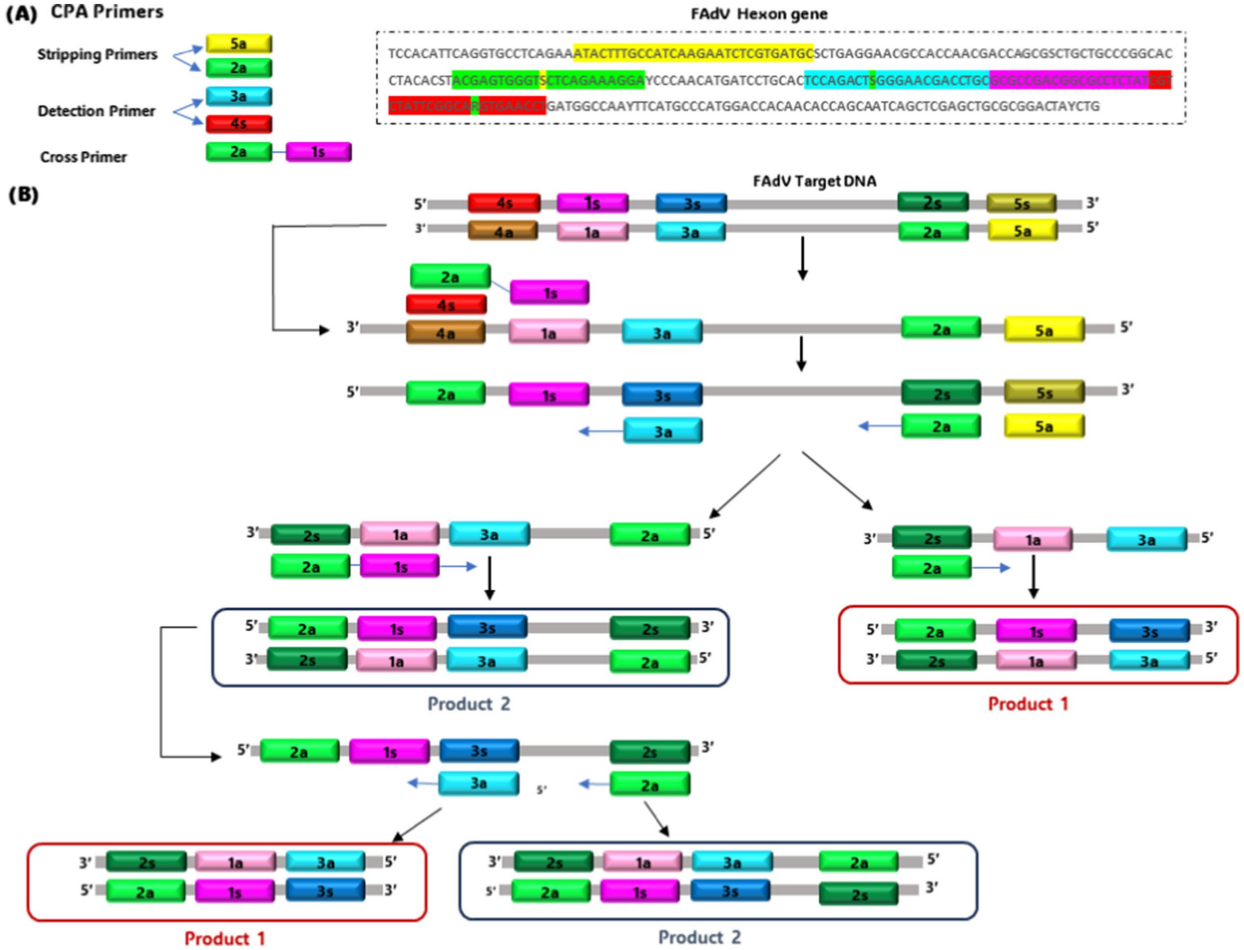
Schematic of single cross-priming amplification technology. **(A)** Primer design site. **(B)** Generation of template with cross-primer sites.

CPA is a technique primarily developed by Ustar Biotechnologies (Hangzhou, China) and initially described for the detection of various pathogens such as Mycobacterium, and Penaeid shrimp white spot syndrome virus. This technique can be divided into single-crossing CPA and double-crossing CPA (84), requiring specific polymerases such as Bst, Bsm, or Gsp SSD (85). Recently, a double-crossing CPA assay has been optimized explicitly for FAdVs detection (86). The assay uses 5 specific primers corresponding to a conserved region of the hexon gene (151-bp) and induces cross-priming amplification with the formation of a hairpin intermediate product (Figure 2). The optimal temperature and incubation time were determined at 68°C for 2 h, respectively. The amplification product was visualized by adding SYBR Green I to the reaction. The test is specific for all FAdV serotypes, and no cross-reactivity was observed with other avian viruses. Its sensitivity was equivalent to that of real-time PCR, reaching 10^−2^ TCID_50_ (TCID_50_: Tissue Culture Infectious Dose). However, the CPA method is faster and cheaper compared to real-time PCR. Consequently, the CPA-FAdV assay has been effectively used to detect 30 field adenovirus strains, representing 7 distinct serotypes (FAdV-1, FAdV-2/11, FAdV-4, FAdV-5, FAdV-7, FAdV-8a, and FAdV-8b) (86).

### 4.3 Recombinase polymerase amplification

RPA is an advanced isothermal technique that was discovered in 2006 by Piepenburg et al. (87). Since the availability of the TwistAmp Basic commercial RPA kit in 2014, the RPA technique has been widely used in the diagnosis of numerous pathogens, such as Human Immunodeficiency Virus 1 (HIV) (88), Ebola Virus (89), Dengue Virus (90), Porcine Circovirus Type 2 (91), Pseudorabies Virus (92), and Foot-And-Mouth Disease Virus (93). The technique relies on the use of T4 phage UvsX recombinase and its cofactor as an essential component that binds to forward and reverse primers (94). The strands are then exchanged after the Single-Stranded Binding Protein (SSB) combines with the parental strand, allowing amplification to continue with the template strand.

**Figure 3:**
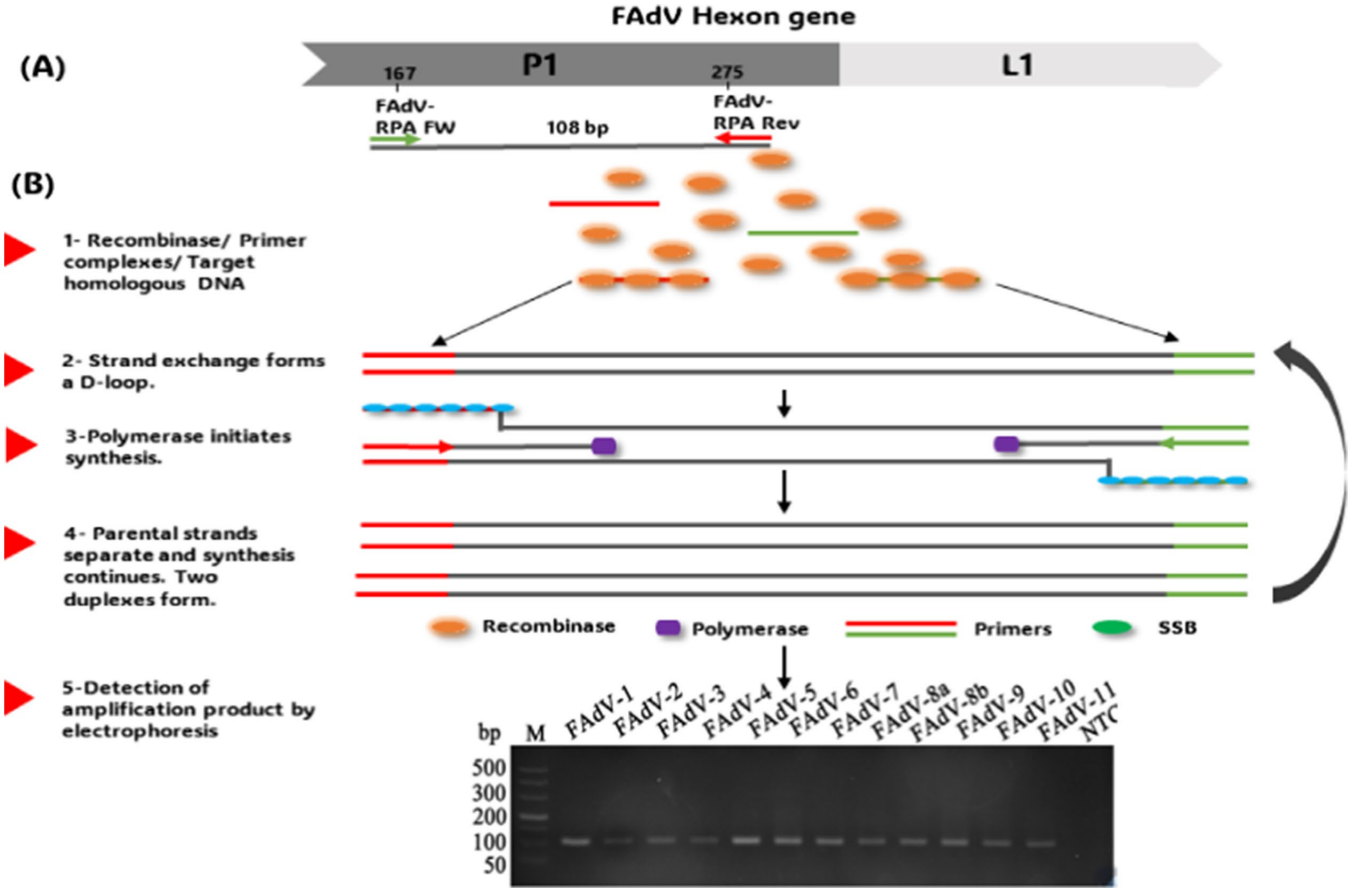
**(A)** RPA primer design. **(B)** Schematic of the recombinase polymerase amplification technique workflow and product detection by Electrophoresis. The gel images presented in this figure are adapted from previous work (101) and are included to ensure the figure is comprehensive and meaningful.

DNA polymerase initiates the synthesis of the template strand from the 3′ end of the primers, forming a new duplex DNA. In this way, a specific fragment is amplified exponentially (95). The amplification occurs between 37 and 42°C (96). However, the amplified signal can be detected by electrophoresis, lateral flow dipstick (LFD), or in real-time using a fluorogenic probe. Real-time RPA and RPA-LFD assays have been developed as attractive and promising tools for rapid, convenient, and reliable detection of *M. ovipneumoniae* in sheep (97), African Fever Virus (AFV) (98), Actinobacillus Pleuropneumonia (99) in swine and Peste Petits Ruminants Virus (PPRV) in small ruminants (100). Recently, an RPA assay has been developed for FAdV detection (101). Primers targeting a conserved region between the 12 serotypes were selected for this assay (Figure 3). Amplification was performed under isothermal conditions (from 26 to 42°C) without using sophisticated thermocyclers in just 14 min. This time is considerably shorter than that of conventional PCR (98 min), while offering similar sensitivity (as low as 0.1 fg viral DNA). However, its sensitivity remains lower than that of real-time PCR (66). The RPA test has revolutionary potential for rapid diagnosis of FAdV. Its rapidity, specificity, simplicity, and adaptability to moderate temperatures make it an ideal technology for large-scale screening of samples, particularly where laboratory resources are limited.

### 4.4 Digital droplet polymerase chain reaction

**Figure 4:**
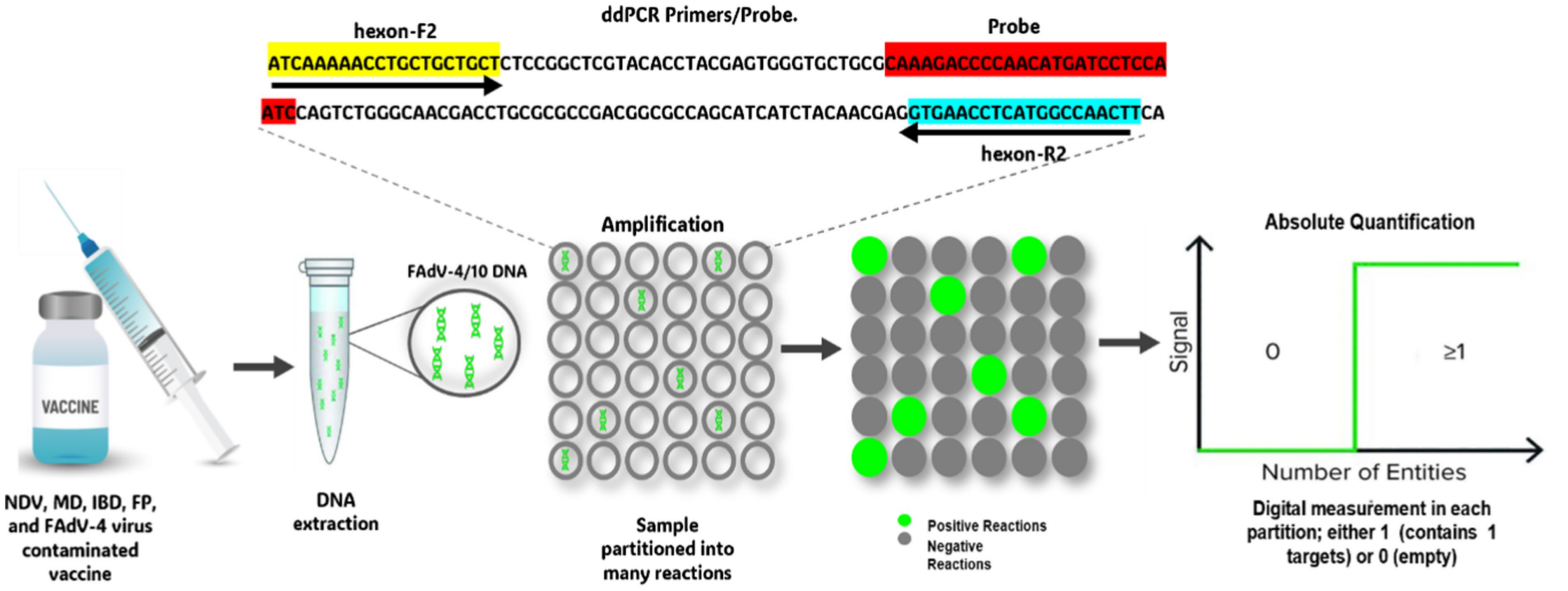
Workflow for detecting vaccine contamination by FAdV-4 using Digital Droplet PCR.

ddPCR represents a significant advance in the precise quantification of nucleic acids, particularly useful in cases where the quantity of DNA or RNA is very low (102). Unlike quantitative real-time PCR, which uses standard curves to estimate the target quantity, ddPCR enables absolute quantification by counting the number of copies present in each sample (103). This method relies on the partition of the PCR reaction into numerous tiny droplets of water in oil. Each droplet contains either zero or a single copy of the target sequence (Figure 3) (104). After PCR amplification, the positive droplets emit a detectable fluorescent signal, while the negative droplets do not. Using statistical calculations, the absolute number of target copies can be determined with great precision (105), highlighting that ddPCR offers exceptional sensitivity and reliability, making it an ideal method for robust quantitative analysis, even in samples with low viral loads or compromised quality (102). This technique has been successfully employed for the precise quantification of defective genomic segments in influenza A virus, providing a highly sensitive approach for detecting these particles in viral stocks (106, 107). Additionally, ddPCR has been applied to detect Chicken Anemia Virus (CIAV) in vaccines, demonstrating its sensitivity in identifying viral contamination (108). Moreover, despite stringent biosafety measures, contamination of live attenuated vaccines with Fowl Adenovirus serotype 4 (FAdV-4) remains a concern, as documented by Yang (109). The use of such contaminated vaccines has been implicated in large-scale outbreaks of Hepatitis-Hepatitis Syndrome (HHS) and Infectious Bronchitis-like Hepatitis (IBH) in poultry populations (110). This is due to the use of vaccines manufactured from chicken embryos with Specific Pathogens Free (SPF), but susceptible to infection by exogenous viruses such as FAdV-4, Avian Leukosis Virus (ALV), and Reticuloendotheliosis Virus (REV) (54, 109, 111). These contaminating viruses can escape detection by most molecular tools, necessitating a highly sensitive detection technique. Consequently, a ddPCR assay has been developed for sensitive detection of FAdV-4 and FAdV-10 in attenuated vaccines (112) (Figure 4). The efficacy of this ddPCR test in detecting FAdV-4 contamination in attenuated vaccines was evaluated in comparison with qPCR and cPCR. Results showed that ddPCR assay could detect FAdV-4 contamination at a concentration of 0.1 EID^50^/1,000 feathers (EID^50^ for Median Infectious Dose), while cPCR and qPCR could detect FAdV-4 contamination at concentrations of 10^2^ EID^50^/1,000 feathers and 1 EID^50^/1,000 feather, respectively. Thus, the ddPCR assay looks 1,000 times more sensitive than conventional PCR detection and ten times more sensitive than real-Time PCR. In addition, the ddPCR assay showed high specificity for FAdV-4/10, generating no positive signals for other FAdVs (112). This makes ddPCR an effective diagnostic technology, particularly for detecting FAdV-4 contamination in live attenuated vaccines. Despite its high cost, the high sensitivity and specificity may contribute to the use of this technique for virus control.

Dot Blot assay is a widely used technique in molecular biology to identify target DNA or RNA.

### 4.5 Dot blot assay combined with cPCR

The Dot Blot assay is a widely used technique in molecular biology to identify a target DNA or ARN fragments with high sensitivity (113). The combination of dot blot with PCR significantly increases test sensitivity (114). Recently, a Dot Blot test has been developed for FAdVs detection (115). The 12 FAdV serotypes have been grouped into 6 categories based on their hexon gene sequence. Subsequently, a conserved region for each category was selected as a probe. Results showed that these probes can efficiently identify the corresponding serotypes, with a detection limit of 10 copies/μL.

Furthermore, the use of a hybrid probe combining all 6 probes at an optimal concentration considerably improved test sensitivity, enabling the detection of one copy of DNA for certain serotypes, which is more sensitive than conventional PCR. The test’s sensitivity was also determined on live attenuated vaccines artificially contaminated with FAdV-4. The results showed that the Dot Blot test can effectively identify exogenous FAdV-4 with an extremely low concentration (1 TCID^50^), whereas conventional PCR can only detect a contaminated vaccine with a viral concentration over 100 TCID^50^ per bottle, demonstrating that the Dot Blot test is 100 times higher sensitivity than cPCR. The same analysis was repeated using vaccines contaminated with mixed serotypes of FAdV, and the same conclusion was reached. In addition, the Dot Blot test was successfully used to diagnose co-infection of FAdV and vertically-transmitted immunosuppressive viruses (CIAV, REV, ALV) in parental flocks with IBH (116). In conclusion, the Dot Blot test, designed based on traditional PCR, is a simple, sensitive, reliable, efficient, and cost-effective tool for the universal detection of all 12 FAdV serotypes.

### 4.6 Nanoparticle-assisted PCR

Nano-PCR is an advanced form of PCR in which solid gold nanoparticles (1 to 100 nm) from colloidal nanofluids are used to improve reaction efficiency, sensitivity, and time (117–119). Compared to other PCR techniques, nano-PCR using nanofluids reaches the target temperature more rapidly, reducing analysis time and nonspecific amplification (120). This technique has been successfully employed in the detection of various viruses such as Pseudorabies virus (121), Porcine Bocavirus (122), Epidemic Diarrhea Virus (123), and Porcine Transmissible Gastroenteritis Virus (124).

Recently, a nano-PCR test has been developed to detect FAdV-4, using primers specific for the FAdV-4 penton gene (125). Test results indicated that nano-PCR has a reasonable specificity, repeatability, and high sensitivity (54 copies/μL), which is ten times higher than that of conventional PCR (cPCR), making it suitable for clinical diagnosis and field surveillance of FAdV-4 infections. Subsequently, a Triplex Nanoparticle-Assisted PCR test has been developed, enabling simultaneous detection of FADV, CAV, and IBDV in one reaction (126). This innovative assay utilizes PCR primers designed to target specific genes of each virus. The test was specific to FAdV, CAV, and IBDV, with a detection limit of 27.2 femtograms (fg) for all 3 viruses’ DNA. This makes it 1,000 times more sensitive than multiplex PCR using identical primers, which provides a simple method for detecting FAdV, CAV, and IBDV infections.

### 4.7 Quantitative PCR refractory amplification (ARMS-qPCR)

**Figure 5:**
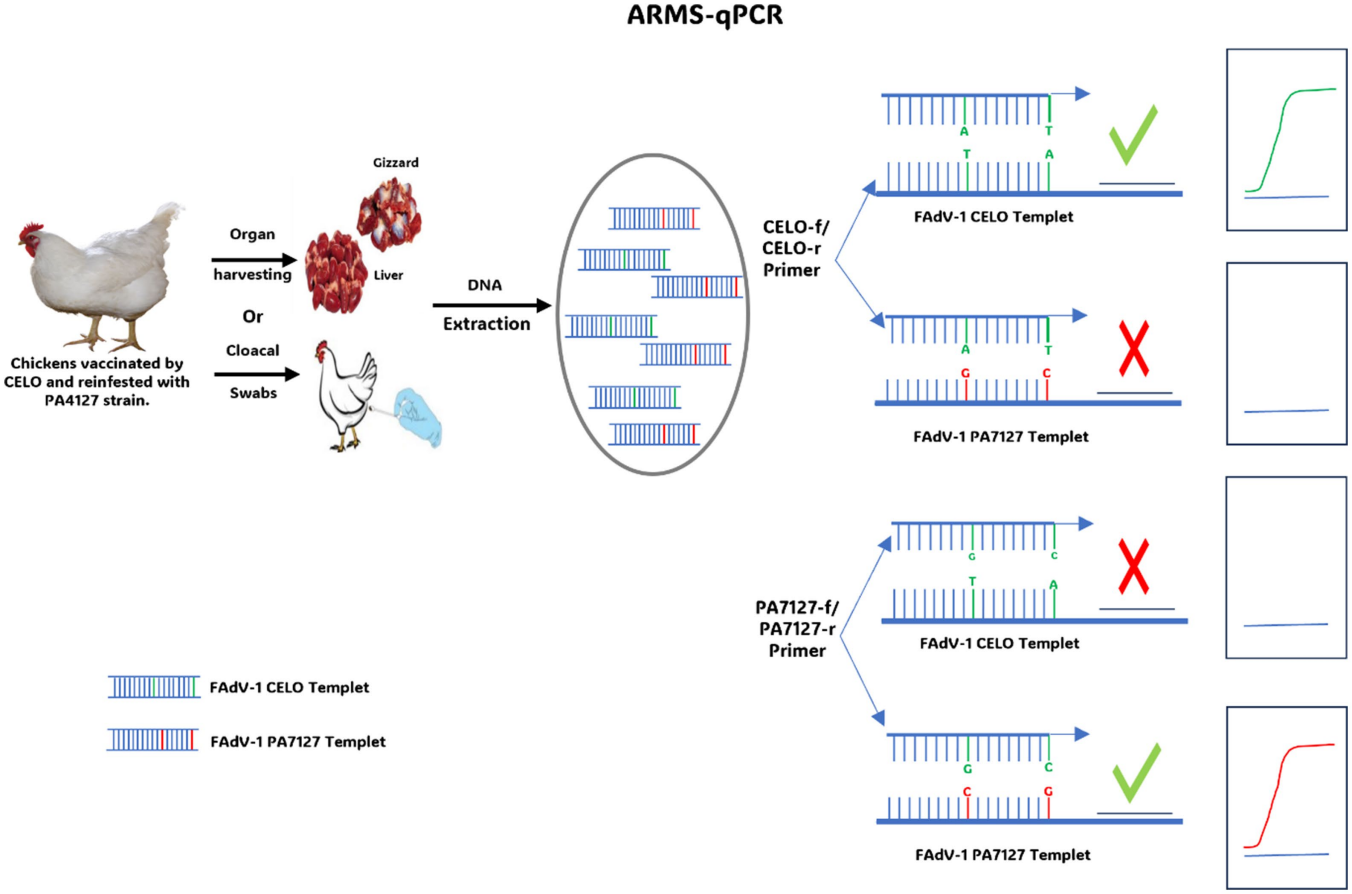
Diagram illustrating the use of ARMS-qPCR to discriminate between the CELO strain and the PA7127 challenge strain.

Quantitative PCR with Refractory Amplification (ARMS-qPCR) also is an innovative molecular tool specially designed to detect and quantify target DNA with high specificity. Unlike other real-time PCR techniques, ARMS-qPCR incorporates refractory amplification, making it highly sensitive and specific for the detection of genetic variations such as Single Nucleotide Polymorphisms (SNPs). This technique is particularly valuable for diagnosing genetic diseases (127, 128) and monitoring the evolution of viruses, bacterial resistance to antibiotics (129), or discrimination between strains of the same genotype based on changes in a few nucleotides (130). In a recent study, ARMS-qPCR was used to quantify and distinguish the European pathogenic strain (FAdV-1/PA7127) from the apathogenic strain (CELO). This distinction is based on SNPs identified in the gene coding for the short-fibre protein (Fiber-2) (131) (Figure 5). Fecal, liver, and gizzard samples from chickens vaccinated with the apathogenic strain (CELO) and challenged with the pathogenic strain (FAdV-1/PA7127) were analyzed by ARMS-qPCR to quantify consensus FAdV-1 DNA as well as FAdV-1 DNA variants (CELO or PA7127). Two pairs of primers, each specific to an FAdV-1 strain, with a hydrolysis probe, were used in this assay. Specificity of discrimination between FAdV-1 strains was ensured using primers targeting SNPs on the 3′ side of each primer. The results confirmed the effectiveness of this test in discriminating between the vaccine and pathogen strains. Furthermore, it was observed that even though chickens were fully protected, they continued to excrete the challenge strain. This observation was achieved for the first-time using ARMS-qPCR. By combining the benefits of refractory amplification with the precision and quantification capabilities of qPCR, ARMS-qPCR represents a powerful method for monitoring vaccines in chicken flocks.

### 4.8 CRISPR/Cas13a-based lateral flow assay

Over the past decade, Clustered Regularly Interspersed Short Palindromic Repeats (CRISPR) associated with Cas (CRISPR-associated) proteins have attracted considerable interest due to their exceptional characteristics, notably their ability to cut DNA with outstanding sensitivity and specificity (132). This has enabled researchers to use it as a molecular scissor for genome engineering (133). CRISPR system is an immune system acquired by bacteria that protects them against viral invasions (134). It works by scanning the DNA of the viral aggressor, degrading it with Cas enzymes, and incorporating segments of the foreign viral genes into regions called “CRISPR Arrays” in the bacterial genome (135, 136). These regions are then transcribed into specific non-coding RNAs (lncRNAs), which direct Cas proteins with endonuclease activity to identify and degrade viral DNA sequences during subsequent reinfections by the same virus (117).

Over the last few years, researchers have discovered that specific Cas proteins, such as Cas13a and cas12a, possess additional cleavage activity, laying the groundwork for a revolutionary new molecular diagnostic method hailed as one of the most impactful “disruptive” innovations of our time (137, 138). By combining CRISPR/Cas13a technology with RPA (CRISPR/Cas13a or cas12a/RPA) (139), this technique has been effectively used to detect AIV (140–142) and other human viruses (143–146).

**Figure 6:**
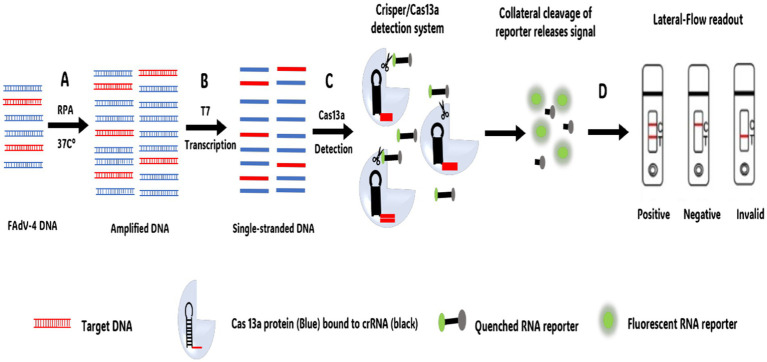
CRISPR-Cas13a DNA combined with lateral flow strips for FAdV-4 detection workflow. **(A)** Recombinase Polymerase Amplification (RPA) performed at a single temperature of 37°C. **(B)** Generation of single-stranded DNA by T7 Polymerase. **(C)** Target sequences complementary to cRNA bind to the CRISPR/Cas13a system. Once the target sequence is present within the system, the non-specific RNA cleavage activity of Cas13a is activated and the reporter RNA is cleaved, resulting in the activation of the fluorescence signal. **(D)** Lateral flow-based detection can be read from strips with a colored positive/negative band using a FAM/Biotin.

Similarly, CRISPR/Cas13a technology, combined with recombinase polymerase amplification (RPA) and lateral flow test strips, has been developed for the rapid, sensitive, efficient, and simple detection of FAdV-4 (147) (Figure 6). This method operates under isothermal conditions at 37°C and enables visual detection through lateral flow strips, with a detection limit of 10 copies/μL. Additionally, a CRISPR/Cas12a assay integrated with LAMP has been developed as a fast, convenient, and cost-effective platform for detecting FAdV-4, offering a detection limit as low as one copy (148) This makes it particularly useful for early viral diagnosis and point-of-care testing.

## 5 Genotyping techniques

Identifying the serotype involved in each case of FAdV infection is essential for understanding pathogenicity, monitoring circulating strains within each country, and developing effective vaccine strategies to control these economically significant diseases (28, 149, 150). Typically, virus typing requires isolation in cell culture or embryonated eggs, followed by a VN test, which was considered as the gold standard protocol for FAdV typing (2, 151). However, this method remains laborious, time-consuming, and requires reference strains and materials. Furthermore, Cross-reaction between serotypes is very pronounced between specific serotypes, which makes results inconclusive (152).

**Figure 7:**
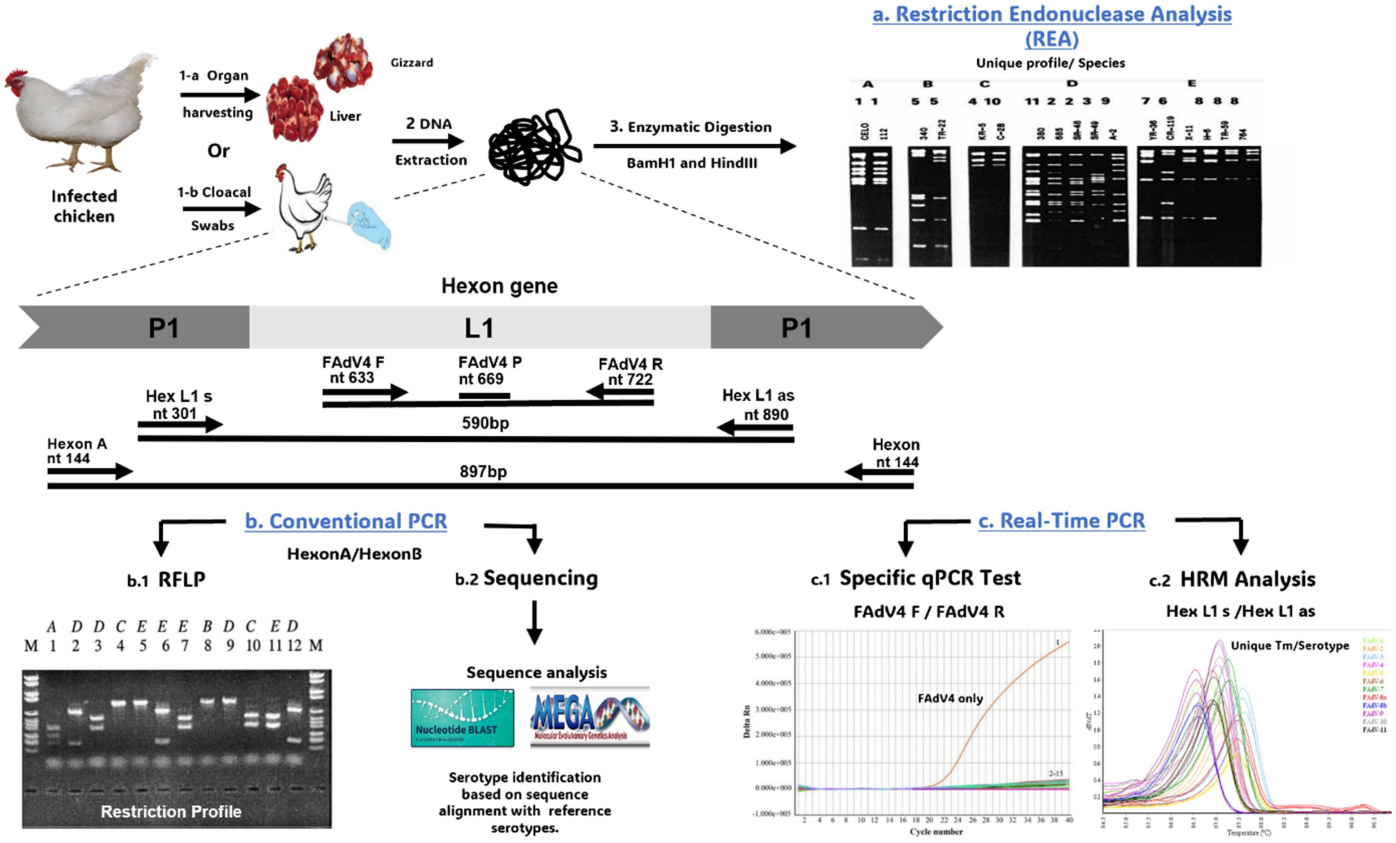
Overview of techniques used for FAdVs genotyping. **(a)** REA digesting the whole genome using BamH1 and *Hin*dIII enzymes. **(b.1)** PCR/RFLP digesting the PCR product derived from HexonA/HexonB primers with restriction enzymes. **(b.2)** Sequencing of PCR product followed by sequence analysis using BLAST and alignment with other FAdVs sequences on GenBank. **(c.1)** Specific qPCR test using a specific primer of serotype (e.g., FAdV4 F/FAdV4 R specific for FAdV-4). **(c.2)** Analysis of High-Resolution Melting (HRM) curve derived from the Hex L1 s/Hex L1 as primer. The gel images presented in this figure are adapted from previous work (43) and are included to ensure the figure is comprehensive and meaningful.

Initially, REA was used to classify FAdVs based on restriction profiles of the whole genome. These restriction profiles, generated by *Bam*HI or *Hin*dIII enzymes, classified 17 FAdV strains representing 12 serotypes into 5 species (Avian Adenovirus A to Avian Adenovirus E) (153). The question of whether FAdV serotypes 4 and 10 should be considered as distinct serotypes or reclassified as a single serotype has been raised (152). This debate stems from the observation of cross-neutralization between serotypes 4 and 10 based on the use of antisera produced in rabbits. Additionally, cross-protection *in vivo* has been detected in chickens vaccinated with the CFA15 strain (serotype 4) and subsequently exposed to CFA20 (serotype 10) through natural infection, demonstrating a strong serological relationship between these 2 serotypes (152). However, whole-genome analysis of both serotypes using restriction enzyme analysis with E.R. *Bam*HI, *Dra* I, *Sma* I, and *Bgl* II has revealed considerable variations (Figure 7).

Since adenovirus neutralization relies on the hexon and fiber proteins, more accurate evolutionary profiles can be expected from the L1 loop rather than the entire DNA restriction profile. It has been reported that the L1 region shows more significant variability than the L2 loop (45). In this context, several conventional PCR assays have targeted the HVR regions of the L1 loop, while restriction enzyme analysis of these PCR products generates serotype-specific restriction profiles (43, 45). Hexon A/Hexon B primers is one of the most widely used primers for FAdV genotyping (43). Subsequent digestion of the PCR product with BsiWI, Sty1, and Mlu1 generates specific restriction profiles for 6 FAdVs serotypes. However, other restriction enzymes must be used to differentiate the remaining serotypes: FAdV-2 and FAdV-11 (Asp1), FAdV-4 and FAdV-9 (Bgl1), or FAdV-7 and FAdV-11 (Sca1).

Nevertheless, one study reported that RFLP using Hexon A/Hexon B failed to classify FAdV-2 and FAdV-11 with the previously mentioned enzymes, and other enzymes must be used (43). Similarly, digestion of the PCR product generated by H1/H2 primer with HaeII differentiated some serotypes, but identical restriction profiles were found for other serotypes. Therefore, we can conclude that multiple digestions using various restriction enzymes might be able to distinguish between 12 FAdV serotypes or that other regions showing high variability between FAdV serotypes should be chosen for RFLP typing.

Besides, PCR product sequencing, generated by Hexon A/Hexon B, followed by analysis using the BLAST bioinformatics tool, is widely used as a reliable tool for genotyping FAdV isolates (see Table 3) (44). However, this technique, like REA and PCR/RFLP, is considered as a multi-step process, making it expensive, time-consuming, and resource-intensive.

**Table 3:** Advanced molecular test for FAdVs diagnosis.

Technique	P forward	P reverse	Probe	Sequences 3′…0.5′	Primer Position	Target gene	Test performance	References
Loop-Mediated Isothermal Amplification.	F2			GTCCCGTCATCACTACTTCG	2405–2424	Hexon	Specific detection of Group I avian Adenoviruses. Rapid detection: 60 min Isothermal amplification in water bath at 63\u00B0C (no thermocycler) Sensitive: detection limit is 238 copies/ul	(81)
		Flc		CACGTCGTGGTCGTACTGGTC	2445–2465			
	Blc			GAGGGCGTGCCTACTTACGC	2493–2512			
		B2		TTGACATTGCTGAGGTCGG	2554–2572			
	F3			TACATGCTGGCGGACATGA	2385–2403			
		B3		CTTGCTGTCCGTTGGTGTA	2577–2595			
	F Loop			GCCTGGTTCCACAGCGC	2424–2440			
		BLoop		TTCCTGCCCGACGGG	2515–2528			
LAMP coupled with a lateral flow dipstick. (LAMP-LFD)	F3			CGTGGCTGAGAGACCTGAT	NR	52 K (FAdV-4)	Specific test for FAdV-4 Rapid detection in 60 min Detection limit is 10 copies/ul 1,000- fold sensitive than cPCR and 100- Sensitive than qPCR	(60)
		B3		TGCACCCCCAAGTCCAG				
	FIP			TCGTGCACACCGCCGATAC				
				CATGATCGTGACCGACCCG				
	BIP			CAAGTTGGCCGCGAAGAAC				
				GCCTGCATCACCCGGTAGA				
LAMP Real-Time Turbidity	F3			AGTCTGGGCAACGACCTG	1681–1698	Hexon (FAdV-4)	Specific to FAdV-4 lower limit detection is 75copies/ul	(82)
		B3		GAATGTTGATGGTGAGGGC	1897–1879			
	F1c			TTACTGGTGTTGTGATCCATGGG	1778–1755			
		F2		CGCCAGCATCATCTACAACGAG	1711–1731			
	B1c			CTGATGCTGAGAAACGCCACC-GG	1789–1809			
		B2		GCACCGAGTATAGAGC	1866–1849			
	LF			AAGTTGGCCATGAGGTTCA	1751–1733			
		LB		GATCAGACCTTCGTGGACT	1813–1831			
Cross-Priming Amplification Method	FAdV-5a			ATACTTTGCCATCAAGAATCTGCT	83–106	Hexon (FAdV-1)	Specific detection of all12 FAdVs serotypes. Sensitivity equal to real-time PCR and bigger to LAMP test. No thermal cycler required Rapid test: reaction time is 2 h. Possibility of differentiating between certain serotypes.	(86)
		FAdV-4 s		AGGTTCACYTGCCGAATAGAC	211–233			
	FAdV-2a			ACGAGTGGGTSCTCAGAAAGGA	130–151			
		FAdV-3a		TCCAGTCTSGGGAACGACCTGC	170–192			
	FAdV-1 s			ACGAGTGGGTSCTCAGAAAGGA	130–151			
				GATAGAGGCGCCGTCGGCGC	193–211			
Recombinase Polymerase Amplification	FAdV-RPA Fw			CKCCYACTCGCAATGTCACCACCGARAAGGCH	NR	Hexon	Rapid detection of the 12 serotypes of FAdVs (14 min) Sensitivity equal to that of cPCR (less than 0.1 fg viral DNA) but inferior to that of real-time PCR	(101)
		FAdV-RPA Rev		TKAHGCTGTASCGCACGCCGRTARCTGTTGGGC				
Dot Blot Assay	P-F-1			CACGCTTCAGCAGGTC	NR	Hexon (FAdV-1)	Specific detection of all 12 serotypes using 6 probes. 100 times more sensitive than cPCR detection of low-dose FAdV-4 (at 1 TCID_50_) in live vaccines	(115)
		P-R-1		GCAGGTAGTCGGCAAT				
			P-1	NR				
	P-F-2-11			CGTCGCCGCTCTTTCA	NR	Hexon (FAdV-2/11)		
		P-R-2-11		AGTTACGCCGCTGGGAG				
			P-2-11	NR				
	P-F-3-9			TTGCGAAAGTTACAGAC	NR	Hexon (FAdV-3/9)		
		P-R-3-9		CCCACGGTTAAGTATG				
			P-3-9	NR				
	P-F-4-10			TTTAACAACTGGTCGGAGAC	NR	Hexon (FAdV-4/10)		
		P-R-4-10		CGATTTCGTAGGAGGGTA				
			P-4-10	NR				
	P-F-5			CCTCCTTCAAGCCCTAC	NR	Hexon (FAdV-5)		
		P-R-5		ACCCGTTCTCCCACA				
			P-5	NR				
	P-F-6-7-8			ACGGCGGCACGGCTTA	NR	Hexon (FAdV-6/7/8)		
		P-R-6-7-8		TCGGGCAGGTAGTCGG				
			p-6-7-8	NR				
Droplet Digital PCR Assay (ddPCR).	Hexon-F2			ATCAAAAACCTGCTGCTGCT	NR	Hexon (FAdV-4/ FAdV-10)	Detection and absolute quantification of FAdV-4 and FAdV-10 in live attenuated vaccines. 1,000 more sensitive than cPCR and 100 more sensitive than qPCR	(112)
		Hexon-R2		AAGTTGGCCATGAGGTTCAC				
			Hexon probe	CAAAGACCCCAACATGATCCTCCAATC				
Amplification Refractory Mutation Systems Quantitative PCR (ARMS-qPCR).	CELO-F:			CGTGTTCAATATGAACCAAAACAT C D	NR		Quantification and differentiation between the two FAdV-1 strains (CELO: apathogenic strain and PA7127: European pathogenic strain) using single nucleotide polymorphisms (SNPs) in the gene coding for the short fiber protein.	(131)
		CELO-R:		AGCCGGTGAAGATAGGCC D				
	PA7127-F:			CGTGTTCAATATGAACCAGAACAC				
		PA7127-R:		CGCCGGTGAGGATAGGCT D				
			P	CCCGAATCGGGAAGCGTAGTAGGG				
High-Resolution Melting-Curve Analyses (HRM).	Hex L1-s			ATGGGAGCSACCTAYTTCGACAT	301–323	Hexon	Differentiation between the 12 serotypes. More efficient than RFLP and VNT.	(50)
		Hex L1-as		AAATTGTCCCKRAANCCGATGTA	890–868			

NR, Not reported; LOD, limit of detection. N = G/A/T/C, M = A/C, R = A/G, W = A/T, S = C/G, Y = C/T, K = G/T, H = A/C/T, D = A/G/T, B = C/.

Recently, a number of studies have shown that real-time PCR combined with High-Resolution Melting Curve (HRM) analysis is a valuable and cost-effective alternative for rapid and efficient genotyping, especially for mass detection, facilitating epidemiological investigations (50, 154). This technique involves the integration of a DNA intercalating fluorochrome into the amplified DNA. The gradual increase in temperature at the end of PCR cycles causes denaturation of the DNA, leading to the elimination of the fluorochrome and resulting in a decrease in fluorescence. These data are recorded by a fluorescence detection system and used to generate a melting curve representing fluorescence changes based on temperature. Slight variations in DNA levels result in variations in the melting temperature (Tm), thus altering the shape of the curve. By comparing the melting curve of a unknown sample with reference curves, it is possible to detect genetic variations, DNA mutations, or even molecular typing (155, 156). HRM technique is widely used in genetic research and molecular diagnostics for its rapidity, sensitivity, and affordability. In 2009, Penelope A. Steer established for the first time a robust closed-tube PCR-HRM genotyping technique for FAdV classification (50). Three primers pairs amplifying 3 different regions within FAdVs hexon gene (Hexon A/B, HexL1-s/Hex L1-as, and HEX-S F/HEX-S R), and generating 3 products of various sizes (897, 590, and 191 bp) were tested.

HRM curve analysis of the PCR product generated by the HexL1-s/Hex L1-as primer proved to be highly sensitive and specific for FAdV genotyping. All serotypes generated one or more significant peaks and were visually distinct from each other in their melting curve profiles, with a confidence level greater than 99%. The applicability of the HRM/PCR Hex L1 assay was also tested on a collection of fields strains from 6 European countries: Pakistan, India, Kuwait, Mexico, Peru, Ecuador, an Australian vaccine, as well as reference strains representing the 12 serotypes, demonstrating that HRM/PCR Hex L1 test is a successful genotyping tool capable of accurately differentiating field isolates from geographically distant regions (50, 154). Subsequently, the PCR/HRM Hex L1 technique was employed to genotype FAdVs from 26 IBH cases in Australian broiler flocks, while cross-neutralization was observed between FAdV-11 and FAdV-2 reference sera using the VN test (157). These findings confirmed that the PCR/HRM Hex L1 assay is a rapid, cost-effective, and more reliable alternative for FAdV genotyping, offering greater accuracy than the VN test, PCR/RFLP, or sequencing for large-scale detection.

## 6 Conclusion

In general, molecular tools play a crucial role in diagnosing and managing FAdV infections in poultry. Unlike conventional diagnostic techniques, real-time PCR has revolutionized FAdV diagnosis, offering highye sensitivity, specificity, suitability for mass detection. In certain cases, more sensitive techniques such as ddPCR are recommended, particularly for detecting vaccine contaminants. For FAdV genotyping, conventional PCR followed by sequencing remains the most reliable method. Additionally, a specific real-time PCR test serves as a valuable tool, enabling detection, quantification, and genotyping in a single reaction, which makes the serotype identification process flexible. However, HRM analysis is an emerging technique that allows the detection, quantification, and identification of FAdV serotypes in a single step, streamlining the diagnostic process and reducing the impact of these infections on the poultry industry. Prioritizing multiplex and highly specific tests for agents involved in conditions like IBH, HHP, and AGE will further streamline the diagnostic process and help mitigate the impact of these infections on the poultry industry.
